# Role of a Digital Return-To-Work Solution for Individuals With Common Mental Disorders: Qualitative Study of the Perspectives of Three Stakeholder Groups

**DOI:** 10.2196/15625

**Published:** 2020-09-16

**Authors:** Patrik Engdahl, Petra Svedberg, Annika Lexén, Ulrika Bejerholm

**Affiliations:** 1 Mental Health, Activity and Participation Department of Health Science Lund University Lund Sweden; 2 School of Health and Welfare Halmstad University Halmstad Sweden

**Keywords:** qualitative method, mental health, mHealth, quality improvement, vocational rehabilitation

## Abstract

**Background:**

Although effective return-to-work (RTW) interventions are not widely available for individuals with common mental disorders on sick leave, there is potential for transforming such interventions into a digital solution in an effort to make them more widely available. However, little is currently known about the viewpoints of different stakeholder groups, which are critical for successful development and implementation of a digital RTW intervention in health care services.

**Objective:**

The aim of this study was to examine stakeholder groups’ perspectives on the role and legitimacy of a digital RTW solution called mWorks for individuals with common mental disorders who are on sick leave.

**Methods:**

A purposeful snowball sampling method was utilized to recruit respondents. Semistructured individual and focus group interviews were conducted for stakeholder groups of service users, RTW professionals, and influential managers regarding their experiences, needs, and preferences for mWorks. Content analysis generated themes and categories that constituted the main findings.

**Results:**

The legitimacy of a digital RTW solution was high among all stakeholder groups since such a tool was perceived to enable service users to take control over their RTW process. This was mainly a product of accessible support and promotion of service user decision making, which had the potential to empower service users. All respondents stressed the importance of fostering a positive user experience with usability and emphasis on service user resources and strengths, as opposed to various limitations and shortcomings. Stakeholder groups highlighted critical content to facilitate RTW, such as the need to clarify a back-to-work plan, accompanied by an accessible RTW network and strategies for handling mental health problems. Implementation challenges primarily involved influential managers’ concern of legislation incompatibility with innovative technology, and RTW professionals’ concern of the possibility that digital solutions may replace them to a certain extent.

**Conclusions:**

This formative research emphasizes the importance of shifting power from RTW professionals to service users. mWorks can play a role in mediating service user control over the RTW process, and thereby increase their empowerment. A digital RTW solution may facilitate the circumvention of implementation barriers associated with introducing evidence-based RTW interventions in a traditional RTW context.

## Introduction

Employment is important for one’s identity, financial security, and sense of involvement in society [1,2]. Common mental disorders (CMD) such as depression and general anxiety disorders are acknowledged as the current leading cause of sick leave and unemployment [3,4]. CMD are associated with an increased risk of extended sick leave (absenteeism), not working at full work capacity (presenteeism), and early retirement [5-9]. Unfortunately, effective return-to-work (RTW) interventions are not widely available for individuals with CMD [10].

RTW can be defined as both a process and an outcome connected to when an individual returns to work after sick leave [11]. In Sweden, RTW is endorsed by different welfare actors (ie, health services, social insurance agency, public employment service, social services, and employers) [12]. However, research has highlighted insufficient collaboration among these actors and employers, contributing to a service and knowledge gap in the RTW process [10,12,13]. Single interventions are performed in a stepwise “train-then-place” manner that are neither coordinated nor integrated into one overall solution to facilitate a person-centered RTW process for individuals with CMD [10,14]. The traditional stepwise approach results in prolonged periods of sick leave and unemployment [15], which in turn negatively impact mental health and well-being [2], empowerment, and the hope and belief that such individuals can work (ie, self-efficacy) [16,17]. During sick leave, individuals often get stuck between mental health services and the next RTW actor [18-20]. In addition to this service gap, there is also a knowledge gap among RTW professionals and employers about how to prevent, recognize, and manage mental health issues [13].

Supported Employment is recognized as the most effective RTW intervention to increase employment among those with severe mental disorders [21] and CMD [22,23], and is distinct from the traditional stepwise approach. Supported Employment is a person-centered, strength-based, and recovery-oriented RTW model characterized by the early introduction of job seeking and rapid placement in employment by a “place-then-train” approach. Thus, instead of performing single interventions in the stepwise and “train-then-place” tradition, Supported Employment is integrated into an overall RTW service corresponding to individual needs [24]. For individuals with CMD, cognitive strategies are included in the Supported Employment approach [22,23]. One Supported Employment intervention is the Individual Enabling and Support model. This model has proven to be more effective in achieving RTW, increasing quality of life, decreasing depression [22], and increasing empowerment [17]. However, effective RTW interventions are not widely available for individuals with CMD due to implementation difficulties, which are largely caused by conflicts between different rehabilitation paradigms when introducing a “place-then-train” RTW approach into a traditional “train-then-place” context [25,26]. In such circumstances, the use of digital interventions that fit the needs of users with CMD have the potential to make RTW interventions more accessible [27,28].

Digital mental health interventions enable service users to gain access to welfare services and interventions regardless of geographical circumstances, time, and place [29], resulting in encouraging user participation and empowerment [30,31]. Some efforts have been made to transform evidence-based, face-to-face interventions such as cognitive behavioral therapy into more accessible mental health interventions such as internet-based cognitive behavior therapy [32]. The effects of these transformations have shown good results in reducing mental health symptoms [33]. These findings motivated the development of a digital RTW intervention called mWorks. The overall mWorks project attempts to transform the Individual Enabling and Support model into a digital solution using mobile phones. To assure its usefulness and implementation, mWorks should be developed in close connection to the implementation context of primary and general mental health services. In particular, understanding of the legitimacy of a complex intervention among stakeholder groups (ie, whether it is recognized as right or acceptable) helps to identify implementation barriers and facilitators before embarking on a lengthy and expensive process of development and evaluations [34]. Therefore, it is critical to address the preferences, needs, and interests of different stakeholder groups at an early stage of development.

The majority of mobile health apps or interventions have not been developed and evaluated in connection to service users’ needs, preferences, and interests [35]. Likewise, it is critical to address implementation challenges at the organization and delivery level of complex interventions [36]. Thus, consideration of the views of different stakeholder groups is important before development of the mWorks intervention to assure that it will become a user- and implementation-friendly digital solution [37]. As a first step, rigorous formative research on service users and other stakeholder groups is required to inform the mWorks design process based on service users’ needs and preferences [35,38]. Second, elucidating potential barriers and success factors that are likely to impact usability, successful development, design, and implementation of mWorks is critical. Accordingly, the aim of this formative study was to gain insight into the role and legitimacy of mWorks, a proposed digital RTW solution for individuals with CMD on sick leave, from the viewpoint of different stakeholder groups, including service users, RTW professionals, and influential people in managerial positions, within the context of primary and general mental health services. A further aim was to inform the development of mWorks.

## Methods

### Design

Formative research helps to identify the needs, preferences, and interests of stakeholder groups that influence usage and delivery. A qualitative descriptive research design with an inductive approach [39] was used to acquire knowledge about the role and legitimacy of mWorks. Ethical approval for the overall mWorks project, of which this study is a part, was obtained from the regional ethics committee in Lund, Sweden (Dnr 2017/324).

### Recruitment and Respondents

Three stakeholder groups were identified (see Table 1). The first group was service users, which include individuals with experience of being on sick leave and of having a CMD such as depression (including depression episodes in bipolar disorder) or an anxiety disorder. The second group was professionals who regularly provide care and support in the RTW process of individuals with CMD, including psychologists, rehabilitation coordinators, physiotherapists, supported employment specialists, occupational therapists, and medical doctors in primary care and mental health services. The last group was stakeholders who held influential, strategic, or managerial positions within the future implementation context. The inclusion criteria for all stakeholder groups were individuals of working age (18-65 years) and able to speak Swedish.

A purposeful snowball sampling method was utilized to find respondents [40]. This sampling method was chosen to find respondents with significant knowledge about the RTW process, the implementation context of primary care and general mental health service organizations, and their digital strategic planning. This allowed for ongoing accrual of new, information-rich respondents who were not known by the study researchers. Initially, the researchers identified stakeholders from each group who had broad connections within the RTW context. A health care strategist in the Skåne County Council was initially contacted by the last author (UB) to identify influential stakeholders. The health strategist was familiar with the RTW research field and knowledgeable about other influential respondents within the organization. Similarly, known RTW professionals within health services were initially contacted, and the first two service users were contacted by the RTW professionals. Each stakeholder was asked about other suitable people who might contribute to the study with valuable information. The snowball sampling method generated participants for both individual and focus group interviews. The choice of interview method was dependent on available resources (time) and existing group affiliation or constellation (eg, psychologists, employment specialists, or service user panel members with experience of CMD and being on sick leave).

**Table 1:** Respondent characteristics (N=46).

Stakeholder group		Age (years), mean (range)	Men/women (n)
**Service users (n=18)**			
	Individual interviews (n=12)	30 (24-48)	7/5
	Service users; one focus group interview (n=6)	55 (44-74)	4/2
**RTW ^a^ professionals (n=20)**			
	Individual interviews (n=12)	44 (30-60)	3/9
	Employment specialists; one focus group interview (n=4)	40 (26-61)	0/4
	Psychologists; one focus group interview (n=4)	40 (38-47)	1/3
**Influential managers in County Council (n=8)**			
	Individual interviews (n=8)	53 (39-59)	1/7

^a^RTW: return-to-work.

### Data Collection

Data were gathered using semistructured individual interviews [41] and focus group interviews (see Table 1) [42]. The interviews aimed to identify stakeholder experiences, needs, and preferences for digital RTW solutions for people on sick leave due to CMD. Interviews were conducted at stakeholder workplaces or at the university of the researchers. The first (PE), third (AL), or last author (UB) conducted the interviews. The semistructured interview guide was based on the questions, structure, and content of a stakeholder study that was similar to the present study, which aimed to develop a digital service in a health service context [43]. The guide contained four topics: (1) earlier experience and interests of digital interventions in an RTW context, (2) perspectives on critical features and content of mWorks to meet the needs of service users, (3) possible obstacles for the implementation of mWorks, and (4) possible success factors. Additionally, probing questions connected to the RTW process were added [41]. The same guide was used for all interviews and stakeholder groups. Prior to the interviews, researchers informed the respondents about the study, and informed consent was obtained from each respondent. Individual interviews were performed by one interviewer (PE, AL, or UB), whereas the focus group interviews were performed by two interviewers with the last author (UB) as the moderator. Each interview was audio-recorded. After each interview, field notes were written by the interviewers, with the additional aim of storing information about the context and setting during the interview.

The intention of the individual interview was to generate a broad range of topics, whereas the intention of the focus group was to reveal additional insight about the respondents’ more sensitive and personal disclosures that are likely to emerge and to allow for discussions about the respondents’ experiences, needs, and preferences for a digital RTW solution. These revelations are more likely to occur in a focus group setting where respondents from a rather homogenous group have the opportunity to explore their group identity and challenge aspects inherent to their subculture, thereby exposing aspects that normally are out of reach in an individual interview setting [44]. Individual interviews lasted 30 to 45 minutes, and the focus group interviews lasted between 45 and 60 minutes. Each transcript was assigned an anonymous code to safeguard respondent confidentiality. The data were stored on a USB drive secured in a fireproof locker at the research facility, with access restricted to the involved researchers.

### Data Analyses

All interviews were transcribed verbatim in Microsoft Word by an independent professional transcriber. The transcribed interviews were initially analyzed inductively by the first author (PE), utilizing Graneheim and Lundman’s [39] framework for conducting qualitative content analysis. The procedure initially involved reading the material several times to gain a sense of the whole content. In the next step, meaning units that corresponded with the overall aim of the study were identified. The meaning units were subsequently condensed into smaller meaning units that still represented the original statement. The condensed meaning units were then coded and organized into categories that illustrated the same phenomenon and represented the manifest content derived from the transcribed interviews. The coding procedure was performed until consensus was reached by three of the authors (PE, UB, AL). Finally, the categories were sorted into themes and a main theme based on knowledge from the literature and the researchers’ professional experience, and these themes constituted the study results. All authors participated in the category sorting. In accordance with manifest content analysis, the level of interpretation and abstraction were kept to a minimum. Throughout the analysis, the focus was to describe the visible and obvious components from the transcripts to best represent what the respondents said in their own words, with exception of the process of establishing themes. The themes presented in Textbox 1 are at a higher level of abstraction than their accompanied categories. To further increase the credibility of the findings, the first author (PE) revisited the raw data in terms of audio files and field notes as well as the transcripts. It was also critical to include citations. Additionally, two different inquiry methods were used to support agreements in findings: individual and focus group interviews [39].

**Subthemes and categories of the main theme that a digital solution enables service users to take control over their return-to-work (RTW) process for individuals with common mental disorders.**

**Supporting service user empowerment**

- Owning one’s RTW process
- Promote decisions with user consent
- Accessible RTW chain

**Addressing implementation challenges**

- Professional attitudes and beliefs
- Legitimacy of digitalization
- Surrounding legislation and policy
- Unforeseen costs

**Create a positive user experience**

- Simplicity
- The importance of design
- Emphasis on resources and strengths
- Alternative communication approaches

**Critical content for return to work**

- Accessible rehabilitation network
- A clear plan
- Strategies for handling stress and anxiety

## Results

### Themes

A main theme and four connected subthemes with categories (Textbox 1) were identified. Overall, stakeholders viewed a digital RTW solution with optimism. The main theme was *a digital solution enables service users to take control over their RTW process.* This theme was derived from the themes indicating that *supporting service user empowerment* may have a positive impact on a digital RTW solution for the service users. Furthermore, respondent statements also elucidated the importance of *addressing implementation challenges* of a digital RTW solution. They perceived implementation barriers to involve personal attitudes among staff, surrounding legislation on a policy level within the organization, and unforeseen costs. Service users thought that it is important to *create a positive user experience* by designing a simple, low-threshold, usable digital RTW solution with an emphasis on service user resources and strengths, and that service users should be able to choose alternative communication approaches. Furthermore, respondents voiced desire for *critical content for RTW,* highlighting the need for an accessible RTW network, clear RTW plan, and strategies for handling stress and anxiety. The findings are represented below by each theme and the accompanied categories (in italics).

### Subthemes and Categories

#### Supporting Service User Empowerment

One of the main positive products of a digital RTW solution that emerged was *owning one’s RTW process.* Respondents (ie, participants from all stakeholder groups) stated that a digital solution would benefit service users in gaining increased control and participation in their RTW process. Service users described the lack of support from mental health services for individuals who want to take control of their RTW process themselves, and believed that mWorks could fill that need. One service user stated:

Well, the mental health service is aimed at those people who are not self-sufficient, those who actually are [self-sufficient] don’t get access to adequate support….But you are still forced to go there while getting worse and worse.

Respondents described the positive aspects of having service users formulate their own authentic plans and goals to achieve RTW. This was thought to create ownership of the RTW process. Influential managers emphasized the potential and importance of a digital solution to empower the service user. By doing so, their own agencies were perceived as becoming more effective, flexible, and accessible. An RTW professional expressed that the service users themselves are the ones who are best informed about their preferences, needs, and interests in relation to RTW. However, some RTW professionals had reservations about a digital tool and cautioned that it could contribute to shifting the responsibility of becoming well or returning to work from the welfare professionals to the service users. They feared that the individual would be left to handle their situation on their own, without the aid of professionals to support them.

Furthermore, respondents thought that a digital solution should *promote decisions with user consent*. A digital RTW solution was perceived as empowering the service user with knowledge and information to prevent decisions made by authorities without service user consent. The ability of service users to lead and control their own RTW process was viewed as positive. The RTW process was described as becoming more transparent with a digital solution, and was otherwise perceived as difficult to grasp and coordinate in traditional services. A digital RTW solution could provide users with knowledge and a voice, while minimizing the potential for authorities to make decisions instead of or without the user, which was considered to be a common process at present. One influential manager of primary care stated: “You would own the process yourself, in the app, and have access to what is needed, and (you) do not have to think about whether something is going on behind your back.”

Influential planners and managers also thought that digital RTW solutions would result in a more *accessible RTW chain*. The threshold for managing the RTW process was anticipated to be lower. Service users described that conventional modes of practice were perceived as rigid and too great of a threshold to overcome. Face-to-face meetings and phone calls were described as stressful to coordinate in a timely manner, but were described as the only viable option for the RTW process. The threshold for contacting the RTW network was described as being lower if there was an opportunity to choose the approach according to individual needs and preferences. Service users validated this perspective by stating that these barriers prevented them from doing anything about their situation. One service user said: “Calling the authorities is something I always try to avoid, because it’s so complicated and difficult.”

#### Addressing Implementation Challenges

The stakeholder groups of influential managers and RTW professionals voiced the need to address implementation challenges. They stated that clinicians or *professional personal attitudes and beliefs* toward digital solutions in general seem to play an important role in the adoption of a digital RTW solution. RTW professionals stated that some of their coworkers perceived challenges and were reluctant to implement new technology. They highlighted that using digital solutions might threaten their ability to keep their jobs, as new and effective work methods could make them redundant. One RTW professional said:

Say that you have a new method, because that means a lot of doubts, if you would create an app that is so good that my job is no longer needed. Then you would not like to support it, would you.

Furthermore, respondents explained how some welfare actors lack the necessary technical skills to utilize new and innovative methods of practice. One service user explained:

The authorities can stand in your way. You have to get them to work together, especially when it comes to technology. I worked with the county council for a while, with their IT department… And that’s terribly bad...So getting them to adapt to ... I believe is one of the biggest obstacles.

The integration of digital solutions could be perceived as an extra workload for which RTW professionals did not have the resources or time. They reasoned that some of their coworkers lacked interest in learning about innovative technology. Individual factors such as attitudes, beliefs, interests, and age were described as important to consider when developing and adopting new technology and the role it might play in their organization. Early voluntary engagement with technology was a predictor for the future willingness to adopt digital solutions. The older coworkers were considered to be less familiar and experienced with technology and how to use it. In contrast, the younger generation was seen as being able to approach digital solutions with greater ease and willingness. The *legitimacy of digitalization* was high among the majority of the influential managers. They felt that a digital RTW solution holds great promise and highlighted the emergence of electronic health and digital solutions as positive, and something they would want to continue to develop in their organization. Influential managers mentioned that the digitalization of welfare services are “the future,” “knocking on the door,” and waiting to be more widely utilized.

Influential managers and RTW professionals expressed that the *surrounding legislation and policy* regulation of privacy and confidentiality made it difficult to use innovative technology in the RTW process. Constant and rapid technological developments made it difficult for legislation and regulations to keep up with technology advances. This was seen as a barrier and one of the reasons potential digital solutions were not fully utilized in their organizations.

*Unforeseen costs* were perceived as a barrier for adopting a digital RTW solution. Service users were afraid that the software would cost money, and thus that they would not be able to afford the app. One service user stated: “That it might be…I don’t know if it should be free of charge, but it would have been good, or at least that it doesn't cost that much.”

Both service users and RTW professionals explained that most of the current mobile apps available were free of charge, and therefore it would be discouraging to pay for an app. RTW professionals noted that not every service user has access to a smartphone since they are expensive. One would need to pay for an internet connection to fully utilize a digital RTW solution, and this was considered to be an additional unforeseen cost for the service user. RTW professionals and influential respondents explained that service users are an economically disadvantaged group and were therefore afraid that expensive software would be a hindrance for adoption.

#### Create a Positive User Experience

The importance of *simplicity* in fostering a positive user experience emerged as an important factor. Respondents emphasized that a smooth, responsive, and fluid user experience, without software bugs, hiccups, and minimal buttons clicks, was important. One RTW professional proposed a “where am I now” function to guide and help the service user orientate and navigate in the app; one should never have to stop and wonder “where am I?” The importance of usability reemerged throughout the data as an important factor for adoption. Excessive information or overly complex configurations were described as cognitively demanding and able to contribute to service user loss of motivation. RTW professionals were concerned that service users would find an overly complex digital solution as overwhelming and an extra workload. They explained that lack of simplicity could generate a loss of interest and adherence, which eventually would result in “dropouts.” Respondents thought that the spoken or written language must be easily understood or should even utilize emojis, symbols, and icons instead of text. This was particularly emphasized by RTW professionals and service users.

Respondents explained *the importance of design* within a digital RTW solution to facilitate continuity and avoid service users’ immediate discontinuation of use. In a focus group discussion about design, an occupational specialist emphasized the value of creating a good first impression: “You have to make a good first impression, I see that as the key to making a successful application—how to make a good first impression.”

Service users wished for universal commands and idioms, utilizing similar design patterns from well-known social media such as Facebook and Twitter. Furthermore, respondents highlighted the importance of being able to adjust the digital solution, in terms of esthetic design, mood, and cognitive ability, to foster individualization. One service user expanded upon the idea of the possibility to alter the degree of simplicity in relation to the capacity or affective state, which may alter from person to person and from day to day. For example, if the user was experiencing cognitive pressure and emotional overload due to stress or anxiety in a particular situation, the need to alter the app for cognitive effort would make it accessible at all times. Service users further explained that the degree of simplicity should vary depending on the stage of their RTW process. Those that were recently on sick leave were perceived as less likely to prefer a complicated app as compared to someone who was about to return to work. One service user explained:

What I feel, when I have been down, when everything is difficult, to go through a mobile with lots of,…lots of settings that I perhaps normally would like….So, when I am down I have no strength for that, …then I would almost like to have it baby-simple.

Furthermore, respondents expressed the importance of *emphasis on resources and strengths* of service users, instead of their various shortcomings and limitations. The need to create a positive user experience with focus on the normality of service users’ conditions, free from judgment and negative reinforcement that might impact the users’ view of themselves, was expressed. One service user articulated the need for a digital RTW solution to be objective and normalizing:

Absolutely. It needs to be very normalizing, I really believe it, because,…because otherwise it is so,…”oh, how ill you are,” so it needs be very like “yes, but this is nothing strange!”

The need for providing *alternative communication approaches* emerged as important for promotion of a positive user experience. Service users observed that conventional means of communication (eg, phone calls and face-to-face meetings) were stressful and anxiety-provoking. The suggestion was that communication be accessible and supported through group chats, text messages, and digital meetings, and the respondents felt positively about these alternative communication approaches. In contrast, some RTW professionals had reservations about fewer face-to-face meetings. They warned that the loss of personal, face-to-face meetings would increase isolation and reduce the amount of social contact among service users. An operating manager from the public employment service stated that RTW professionals viewed face-to-face meetings as superior to alternative communication approaches: “We must be aware and reconciled about our overconfidence in face-to-face meetings, which we often believe to be superior in comparison to digital meetings.”

Respondents highlighted the benefits of introducing more viable communication options within their RTW network and noted progress toward RTW in a manner that felt suitable and comfortable to service users. They articulated the desire to be able to digitally record and retain documentation from RTW meetings. They often perceived meetings as stressful and indicated that it was difficult to register all of the information. Service users experienced being misinterpreted as lazy when they had difficulty understanding and remembering what was said during the meetings because of stress. As a consequence, one respondent explained being discharged by the psychiatrist:

People always assumed I didn’t care, but I was just misunderstood. I've always cared, but they thought: “She doesn’t care, why should we help her?”

#### Critical Content for RTW

A wide variety of content was suggested as facilitating RTW for service users. An *accessible rehabilitation network* was considered to be paramount. Respondents explained that this network usually involved welfare actors who were assigned to support the service user until employment, such as occupational therapists and physiotherapists, supported employment specialists, social workers, medical doctors, handling officers at the national social insurance agency and public employment service, as well as family members, spouses, or friends. Service users proposed that contact information could be available to provide shortcuts in their RTW network. Having quick access to the rehabilitation network and to certain RTW professionals was perceived as an advantage that could prevent stress and anxiety-provoking scenarios. One service user said:

The reason why I think it must be as quick as possible, is...because of stress….The cause of stress is, of course, that it does not go (away) fast enough. Another reason is that you do not have anything to do, but…if you are on sick leave for one or another reason, just waiting is the most dreadful thing that exists. That’s another negative thing that can happen, and so you have to solve it immediately. It can occupy your thoughts a whole week until you have solved it.

Respondents perceived that access to the RTW support network was limited and inefficient due to travel distances. An accessible RTW support network was described as fast, with efficient means of communication regardless of geographical circumstances. Service users conveyed that an accessible RTW support network contributes to a sense of safety, because they know that they have access to support if needed. However, a psychologist raised concerns about being available around the clock. They suggested that the RTW professional network should only be available during working hours.

To create *a clear plan* for the service users was commonly recommended regardless of the stakeholder group. The importance of a calendar, schedule, and reminder features was emphasized. The building blocks or strategies for back to work need to be clear. The service user position or stage in the RTW process needs to be located, and the important goals or steps that need to be taken, along with the appropriate strategies to carry out at each step must be well-defined. In conjunction, a “to do list” that illustrates needed actions was suggested. RTW professionals, especially psychologists, proposed these kinds of functions to enhance motivation by establishing feasible and meaningful milestones and goals. Thus, users could measure and monetarize their own progression through a clear RTW plan.

*Strategies for handling stress and anxiety* were proposed as important. Functions to support coping with anxiety and stress when such emotions arise at the workplace or everyday life were stressed, mainly by service users and RTW professionals. Features like mindfulness, cognitive behavior therapy strategies, and relaxation or recovery exercises were recommended. One influential manager for a primary health care facility proposed that the RTW solution could contain a “first-aid kit” with personalized strategies to cope with stressful and anxiety-imposed scenarios. Respondents thought that there should be interactive functions for access to fast and reliable information about service user symptoms and problems. Service users explicitly wished to understand their thoughts and emotions when stress and anxiety arose. They proposed links to external webpages with reliable sources of information.

## Discussion

### Principal Findings

These findings show that a digital RTW solution has a role to play in the RTW process, and has legitimacy among stakeholder groups. A wide variety of factors need to be considered as an important precursor of the development of mWorks. The primary finding is the importance and capacity for mWorks to foster service users’ control of their RTW process. According to the themes, a digital RTW solution that can satisfy stakeholders and will enhance service user empowerment needs to be developed in relation to existing implementation challenges, while fostering a positive user experience and focusing on the different stages and parts of the RTW process.

All stakeholder groups favored empowering service users and agreed that the forthcoming mWorks should promote conditions for service user participation and ownership of their RTW process. This same theme emerged in recent qualitative research on digital solutions [45,46]. According to the respondents, regardless of group affiliation, one way of promoting such conditions would be to lower the threshold for service users to manage and control their RTW process, irrespective of their mental health. Our findings also suggest that mWorks needs to focus on making the user RTW support network accessible, regardless of time, place, or pace [29]. Previous research has identified having an overview of critical RTW actors and professionals as a critical RTW factor [47] that makes the service user more informed and in charge of the RTW process. Thus, mWorks has the potential of increasing service user empowerment along with their sense of control over the different RTW steps and the RTW support network, which has previously been inaccessible or difficult to comprehend. Our research elucidated several implementation barriers that might be encountered with a digital RTW intervention.

Although legitimacy was high among all stakeholder groups, managers raised concerns about the legislation and policy regulations of privacy and confidentiality. These circumstances are likely to impede implementation of novel digital interventions if not accounted for during the design and implementation process. There are implementation challenges at several levels that influence the RTW process, not only aspects at the individual level but also those related to legal or organizational structures. In a systematic review, Powell and colleagues [48] stressed the importance of addressing barriers associated with implementing mental health service interventions at multiple levels within the implementation context. On an individual level, our findings suggest that stakeholder attitudes and beliefs toward digital RTW solutions may constitute a barrier. For instance, RTW professionals indicated negative biases toward digital RTW interventions because of a lack of time, resources, interest, and the potential threat of job loss. The latter has been found in earlier qualitative research, in which mental health service staff reported reluctant attitudes toward digital interventions due to the potential for them to replace clinical care [49]. Addressing professionals’ views on digital solutions is important to foster successful adoption and implementation of such interventions [29,46]. These conservative stances suggest the need to highlight the added benefits (ie, increased effectiveness, and flexible and accessible support [30]) for both service users and RTW professionals when incentivizing digital RTW solutions. Rather than replacing face-to-face interventions in health care services, Berry et al [46] reported that a digital solution can enhance existing support. Therefore, a digital RTW solution should be understood as complementary to traditional support rather than replacing it.

Another implementation barrier pertains to reliance on access to a mobile device and internet connection. While it is true that service users are an economically disadvantaged group [50,51], research shows that individuals with severe mental disorders have almost as much access to mobile devices as the general adult population. Although it seems reasonable to assume that individuals with CMD may have a better financial situation than those with severe mental disorders, one of the most common barriers for mobile device ownership is the monthly subscription plan expenses [52]. Previous research had suggested discount programs to address the affordability of digital solutions for service users [53].

With regard to usability, our findings highlight the need to design a simple digital solution that fosters a positive user experience for individuals with CMD who may have a lack of motivation or difficulties in comprehending information. Some of the service users thought that complex digital interventions are likely to generate a lack of engagement. Our research emphasizes the importance of a focus on user strengths and resources rather than on problems and shortcomings. Comparable results were found in qualitative research when respondents stated that digital solutions need to foster positive feelings, without focusing on the negative aspects of CMD and symptoms that could lead to ruminating and catastrophizing [46]. To assure that mWorks promotes a positive user experience, service users must be included in the inquiry and design process. Users will not enjoy or adopt products that focus on their limitations, but they are capable of suggesting ways to reduce focus on the negative aspects of CMD [54]. User-centered research with a participatory, iterative design should be employed to ensure that mWorks is grounded in service user preferences that enhance their strengths and resources. Participatory design is compatible with the Individual Enabling and Support model, which focuses on individual preferences and needs [24], and further validates the need to make the Individual Enabling and Support model more accessible through a digital solution. To create a positive user experience, the introduction of mWorks should be paired with informational or educational efforts to help service users get started and thus minimize the risk of their immediate termination of use [55]. The app’s digital pedagogical presentation, and how that is understood by the user, should also be considered. These findings highlight the importance of introducing digital solutions that are attuned to individual RTW needs and preferences, as well as the need for pedagogic structure and information on usage.

According to the stakeholders, the role and legitimacy of a digital RTW solution are associated with having access to adequate RTW support, regardless of time or place. In addition, the content should help service users gain a clear overview of the RTW environment. The development of mWorks might make the RTW steps more visible and tangible for service users. The opportunity to make a clear, individualized plan of how to get back to work, mediated through a schedule or “to do” list, can provide a setting with feasible goals. This kind of goal setting has been shown to generate increased levels of self-efficacy [56]. In turn, self-efficacy is one of the most important determinants for RTW [57,58]. Using goal-setting strategies to establish meaningful goals could help service users manage and prioritize their next appropriate step toward RTW.

Another way to help service users establish and reach their goals would be to borrow from motivational theories [59]. Similar suggestions have been mentioned in earlier research about how to help users set and reach goals through increased motivation and engagement [35,60-62]. Motivational interviewing can be successful in helping people identify their goals [63] and RTW [24]. Bakker et al [35] suggested the value of self-determination theory in the development of a digital solution that would increase service users’ intrinsic motivation. Another proposal to enhance goal achievement would be to utilize game elements. The use of gamification has shown promising results in research that used goal setting theory to increase engagement [63-67].

Our findings highlight the importance of including cognitive strategies in mWorks to cope with stress and anxiety at work and in everyday life. Doing so would generate a sense of safety since users would have access to cognitive strategies and their RTW support network regardless of geographical circumstances. However, the service user group warrants a swift but reliable contact with professionals in their RTW network, as opposed to one psychologist who expressed concern about the need for psychologists to be available to service users around the clock. This concern emphasizes the importance of making cognitive strategies accessible outside of office hours. Internet-based cognitive behavioral therapy is an effective strategy to address stress and anxiety [32,33], and can be fully delivered as automated conversational apps that foster self-management [68]. These cognitive components can serve an important role in promoting an mWorks service user who manages and controls their RTW process.

### Methodological Considerations

We used the Consolidated Criteria for Reporting Qualitative Research guide [69], which is a 32-item checklist to ensure the quality of the study. Carrying out formative qualitative research with stakeholder groups throughout the design and development process has been identified as an important cornerstone to tailor digital interventions to service users’ needs and preferences [70]. To enhance the transferability (ie, external validity) of the findings, we took field notes, which allowed for a more in-depth description of the research setting and data collection procedure [71]. Purposeful snowball sampling was considered to be a desirable method of recruiting interview respondents because the researchers had no previous insight into potential respondents. Nevertheless, the snowball sampling method can be criticized for skewing the sample in a specific direction [41]. To increase transferability, the researchers asked the respondents to suggest only one or two potential respondents per person per stakeholder group. The utilization of individual and focus group interviews was selected to allow for a wider variety of data to emerge so as to not rely on only one source of inquiry. This helped the researchers reach adequate saturation and enhance the credibility of the findings [71]. Individual interviews are highly effective at generating a broad range of topics, while focus group interviews are more likely to produce sensitive and personal disclosures [44]. The respondents did not get the opportunity to check the transcripts or the interpretations, which negatively affects the credibility and is a limitation of the current study [71].

The authors constitute an interdisciplinary research team with expertise in their respective research fields, including public health (PE); digital development and participatory research (PS); occupational therapy, CMD, and RTW in relation to service users, employers, and RTW professionals (AL, UB); and mental health services and implementation research (UB). UB created the project idea about translating the Individual Enabling and Support model into a digital format called mWorks. Additionally, the first author (PE) has knowledge of the RTW process through his own experience of sick leave and CMD. This contributed to a healthy mixture of perspectives during the analysis process, and minimized the chances of having personal biases influence the findings. This in turn enhanced the credibility (ie, internal validity) of the findings, and therefore increases the level of trustworthiness of the current study [39].

### Conclusions

mWorks may facilitate the avoidance of conflict between different RTW paradigms. This conflict has been a major implementation barrier of introducing a “place-then-train” model in a “train-then-place” RTW context [25,26]. Shifting the power from health care professionals to the service users is a clear priority [72,73]. Service user empowerment is emerging as a focal point in mental health research and reforms, but the understanding of how to implement such a paradigm shift is still underdeveloped [74]. mWorks may have a role to play in such a paradigm shift. Further research should focus on conducting user-centered research with a participatory iterative design to best understand service user needs and preferences when developing digital RTW solutions.

## Abbreviations

CMD: common mental disorders
RTW: return-to-work
